# The Genetic Basis of Pericentral Retinitis Pigmentosa—A Form of Mild Retinitis Pigmentosa

**DOI:** 10.3390/genes8100256

**Published:** 2017-10-05

**Authors:** Jason Comander, Carol Weigel-DiFranco, Matthew Maher, Emily Place, Aliete Wan, Shyana Harper, Michael A. Sandberg, Daniel Navarro-Gomez, Eric A. Pierce

**Affiliations:** Ocular Genomics Institute, Berman-Gund Laboratory for the Study of Retinal Degenerations, Massachusetts Eye and Ear Infirmary, Harvard Medical School, Boston, MA 02114, USA

**Keywords:** pericentral, retinitis pigmentosa, pericentral retinitis pigmentosa, pericentral retinal degeneration, genotype/phenotype correlations, rhodopsin, *HGSNAT*

## Abstract

Pericentral retinitis pigmentosa (RP) is an atypical form of RP that affects the near-peripheral retina first and tends to spare the far periphery. This study was performed to further define the genetic basis of this phenotype. We identified a cohort of 43 probands with pericentral RP based on a comprehensive analysis of their retinal phenotype. Genetic analyses of DNA samples from these patients were performed using panel-based next-generation sequencing, copy number variations, and whole exome sequencing (WES). Mutations provisionally responsible for disease were found in 19 of the 43 families (44%) analyzed. These include mutations in *RHO* (five patients), *USH2A* (four patients), and *PDE6B* (two patients). Of 28 putatively pathogenic alleles, 15 (54%) have been previously identified in patients with more common forms of typical RP, while the remaining 13 mutations (46%) were novel. Burden testing of WES data successfully identified *HGSNAT* as a cause of pericentral RP in at least two patients, suggesting it is also a relatively common cause of pericentral RP. While additional sequencing might uncover new genes specifically associated with pericentral RP, the current results suggest that genetically pericentral RP is not a separate clinical entity, but rather is part of the spectrum of mild RP phenotypes.

## 1. Introduction

Retinitis pigmentosa (RP) is well known for being a genetically heterogeneous disease, with mutations in at least 89 different genes known to cause nonsyndromic RP alone [1]. As mutations in so many different genes can cause essentially the same phenotype, this makes RP one of the most genetically heterogeneous diseases in humans. However, while RP is usually characterized by typical “bone-spicule” pigmentation and photoreceptor degeneration beginning in the mid-peripheral retina [2,3,4], it would be an oversimplification to say that all RP phenotypes are the same; several subtypes of RP have been clinically defined, including pericentral RP, sector RP, pigmented paravenous RP, and RP without pigment [5,6,7,8]. It remains to be determined to what extent these clinical subtypes stem from different genetic causes, or whether they are, for example, a reflection of variable expressivity of phenotypes due to mutations in the same set of genes [3]. This study aimed to address this question by expanding the search for genetic causes of a particular subtype of RP—pericentral RP. We further hypothesized that by studying a cohort of patients with an atypical phenotype, it would increase the possibility of uncovering new biological pathways or genes involved in RP.

Pericentral RP has been described as a subtype of RP in which, instead of the pathology starting in the mid-periphery like typical RP, the disease starts in the near periphery closer to the vascular arcades and tends to spare the far periphery. As this is a clinically defined phenotype, the definition of pericentral RP and even the name of the condition can vary somewhat between authors. Similar phenotypes have been described as: pericentral pigmentary retinopathy [9,10,11], pericentral pigmentary retinal degeneration [12,13,14,15], pericentral retinal dystrophy [16], peripapillary retinal degeneration [14,17], perivascular retinal pigment epithelium atrophy [18], pericentral retinal degeneration [19], RP with perimacular or paramacular pattern [2], and pericentral RP [20,21,22]. A natural history study from our institution defined the phenotype as bone-spicule pigmentation or atrophy in the near mid-periphery corresponding to an annular scotoma from 5–30 degrees, a normal or nearly normal dark adaptation threshold, and subnormal but readily detectable full-field electroretinogram (ERG) responses [22].

The genetic causes of pericentral RP have not been fully defined to date. The pericentral RP phenotype has been found in both dominant and recessive pedigrees [10,16,18,19]. Three families with pericentral phenotypes were found to have mutations in rhodopsin (*RHO*) [23]. Further studies, specifically of the phenotypes of patients with *RHO*-associated RP, revealed one subset with pericentral defects [24]. Mutations in the *TOPORS* gene have been reported to cause a pericentral RP-like phenotype in two pedigrees [18,25]. In those studies, the phenotype was termed “pericentral retinal dystrophy” or “RP with perivascular retinal pigment epithelium atrophy” [18,25]. Another study reported two patients with *TOPORS* mutations that caused typical autosomal dominant RP without the presence of a perivascular cuff of retinal pigment epithelium atrophy [26]. Further putative mutations in *TOPORS* in a panel of RP patients have been reported [27]. The largest study of pericentral RP genetics to date identified the molecular cause of disease in 14 of 28 pericentral cases [19]. The most commonly identified genes among the 14 solved cases were *ABCA4* with 5 cases and *CERKL* with 2 cases. The authors concluded that there was molecular heterogeneity in the pericentral phenotype, making it an uncommon phenotype composed of many different genotypes. They noted the genes involved have also been associated with other phenotypes, such as maculopathies and typical RP.

One non-genetic cause of a pericentral RP-like phenotype is a form of hydroxychloroquine (Plaquenil) toxicity; the pericentral pattern of pathology (as opposed to the usual perifoveal type) is more common in Asian patients [28,29]. None of the patients described in this study are known to have a history of hydroxychloroquine use.

We performed panel-based sequencing and whole exome sequencing (WES) of a larger cohort of patients with pericentral RP to better define the genetic causes of this phenotype. In addition to extending the observation that the genetics of pericentral RP show a diversity of genotypes, the results obtained from this study indicate that the genetic causes of pericentral RP are similar to those that can cause mild versions of typical RP. Using WES, *HGSNAT* was also identified as a recurring cause of pericentral RP.

## 2. Materials and Methods

### 2.1. Cohort

We identified a cohort of 45 probands with pericentral RP based on a comprehensive analysis of their retinal phenotype. They underwent a comprehensive evaluation in the Electroretinography Service at the Massachusetts Eye and Ear Infirmary, including best corrected visual acuity, Goldmann visual field tests with V4e and I4e test lights, final dark-adapted threshold with an 11 degree test light in the Goldmann-Weekers dark adaptometer, and full-field ERG. ERG responses included a white light mixed response measured at 0.5 Hz and a cone flicker response measured at 30 Hz, as previously described [30]. Inclusion in the cohort was determined by the clinical diagnosis of an experienced physician (E.L. Berson, Massachusetts Eye and Ear Infirmary, Massachusetts, MA, USA) based on the above testing. In summary, factors included bone-spicule pigmentation or atrophy in the near mid-periphery (5–30 degrees) corresponding to an annular scotoma, a normal or nearly normal dark adaptation threshold, subnormal but readily detectable full-field ERG responses, and a healthy-appearing anterior retina [22]. Not all findings were present in every patient. The phenotype has been described in depth in previous studies [19,22]. For further references please see the introduction.

Blood samples were collected for leukocyte DNA. DNA samples were collected from 45 probands, along with affected and unaffected relatives when available, for a total of 61 samples. To the best of our knowledge, none of the probands were related. The study protocol adhered to the tenets of the Declaration of Helsinki and was approved by the Institutional Review Board (Human Studies Committee Protocol 11-057H, approved 2012-present) of the Massachusetts Eye and Ear Infirmary.

### 2.2. Panel-Based Exon Sequencing

A custom SureSelect targeted enrichment kit (Agilent, Santa Clara, CA, USA) was designed to capture and analyze the coding regions and untranslated regions (UTRs) of 196 genes known to cause inherited eye disease [31]. Later versions tested 226 genes. Libraries were generated using standard methods [31]. This Genetic Eye Disease (GEDi) panel was analyzed with a MiSeq platform (Illumina, San Diego, CA, USA) using 2 × 121 bp reads, multiplexing 9 to 12 samples per run.

Panel-based sequencing data was analyzed as previously described [32,33]. Briefly, sequences were aligned with BWA [34], and SAMtools [35] was used for duplicate removal and variant detection. Variants were annotated and filtered using internal data as well as publicly-available sources. Variant types that were considered included nonsynonymous changes (i.e., protein sequence altering changes), splice-site changes, or variants previously described as disease-causing in the Human Gene Mutation Database (HGMD) [36] or Clinvar [37]. An in-house, web-based variant browsing tool aided browsing and collation of results. Initial allele frequency cutoffs were set at 1/10,000 for dominant disease and 1/700 for recessive disease based on similar values for the most common alleles that cause RP. Standardized variant names were validated using the online tool Mutalyzer (https://mutalyzer.nl/).

### 2.3. Sanger Validation

Mutations detected by next-generation sequencing (NGS) were sequenced using Sanger sequencing. Genomic regions of interest were PCR amplified with optimization of cycling temperatures, purified (ExoSap-IT, Affymetrix, Santa Clara, CA, USA), and sequenced (BigDye Terminator v3.1, ABI 3730xl, Life Technologies, Grand Island, NY, USA).

### 2.4. Copy Number Variation (CNV) Analysis

Nine patients without disease-causing mutations identified using targeted exon sequencing then underwent CNV analysis using Omni2.5 chips (Illumina) according to manufacturer instructions. Regions with potential deletions or duplications were identified using CNV Workshop [38].

### 2.5. Whole Exome Sequencing and Burden Test

Targeted enrichment was performed using the SureSelect XT Human All Exon + UTR v5 baits (Agilent). Libraries were sequenced on an Illumina HiSeq platform using 2 × 101 bp reads in a 16-sample multiplex. Variants were identified using the pipeline described above for panel-based sequencing. Variants of interest were also validated using a pipeline based on BWA alignments and Genome Analysis Toolkit (GATK) joint variant calling, following Broad Institute best practices [39]. An automated gene “burden” analysis [40] was conducted to detect genes where predicted loss-of-function mutations are overrepresented in the unsolved pericentral cohort compared to all other exomes run at our institution. The pericentral cohort consists of 16 samples that were not initially solved using panel-based sequencing (except #24, which had both panel and exome sequencing). The test looks for overrepresentation, on a gene-by-gene basis, of damaging variants in the 16 unsolved samples, as compared to the remainder of the WES sample cohort. A variant was considered “damaging” if it was an exonic, non-synonymous variant which is: either a frameshift insertion or deletion (i.e., length not divisible by 3), a stop-gain mutation, a splice variant at ±1 or ±2 locations, or predicted as damaging by either PolyPhen [41] or SIFT [42]. Synonymous and UTR variants were not included. Variants seen >20 times in the entire exome dataset were discarded as too common to be a cause of a rare Mendelian disease. The number of variants meeting these conditions was counted on an allelic basis in each sample (e.g., autosomal homozygous calls counted as 2, heterozygotes as 1). First, a recessive model was applied, which required a damaging homozygous or compound heterozygous variant for a sample to be counted. Separately, a dominant model was applied where only one damaging heterozygous or homozygous variant had to be present to be counted. Then, for each gene in the dataset, a right-sided Fisher test was computed to assess the overrepresentation of samples with damaging variants in the pericentral cohort versus all other exomes. All genes were then ranked by their resultant Fisher scores to identify genes whose damaging variants have maximal over-representation in the pericentral cohort.

## 3. Results

### 3.1. Cohort

**Figure 1:**
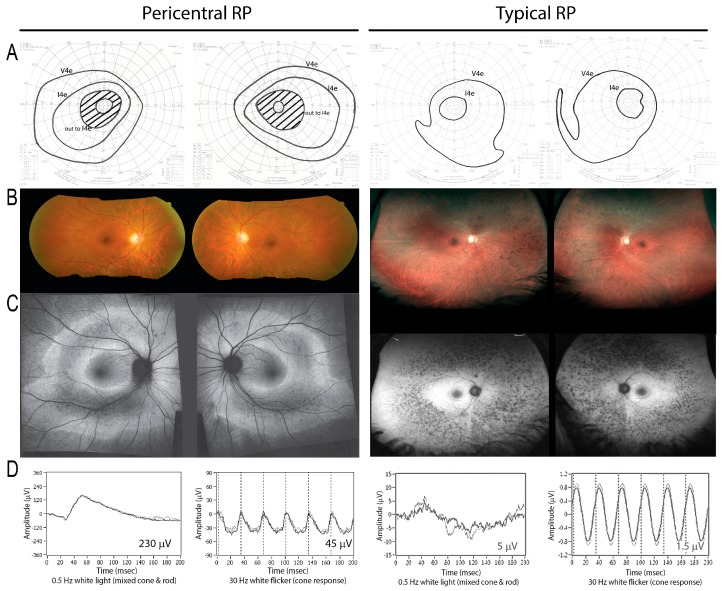
On the left, a patient (Family #14) with a heterozygous *RHO* mutation demonstrates a pericentral phenotype, with pericentral scotomas but preserved peripheral field to the I4e stimulus on visual field testing (**A**). There is retinal pigment epitheium (RPE) atrophy in the near periphery as shown by fundus photo (**B**) and autofluorescence imaging (**C**), as well as relatively preserved electroretinograms (ERGs) (**D**). In contrast, in typical retinitis pigmentosa (RP) (right), the peripheral response to I4e is lost (**A**), and the affected area is located farther away from the macula in the mid-periphery (right, **B**,**C**; note lower magnification). The ERG is more severely affected (**D**; note different scales; see numerical values, inset).

Baseline clinical characteristics are shown in Table 1. An example of how the pericentral RP phenotype differs from typical RP is shown in Figure 1.

### 3.2. Panel-Based Sequencing Results

The panel-based NGS approach (GEDi panel [31]) provided a mean depth of coverage of the targeted sequences of >100-fold, and >97% of the targeted sequences were covered with ≥10× depth. Out of 43 families, 44% (19 families) were solved genetically by identifying mutations provisionally responsible for disease (Table 2). Within those 19 solved families, there were: 7 dominant, 1 X-linked carrier, 2 homozygous recessive, and 9 compound heterozygous recessive. Therefore, there were a total of 28 (7 + 1 + 2 + 18) putatively pathogenic alleles. Fifteen of these 28 alleles (54%) have been previously reported in patients with typical RP or an RP-associated syndrome, while 13 alleles (46%) are novel. See supplemental Table S1 for additional annotation including genomic coordinates, pathogenicity predictions, pedigree type, references, and notes discussing the unsolved/partially solved patients with variants of unknown significance (VUS).

All variants that were validated with Sanger sequencing showed the expected results (Table 2). During the course of this study, in-depth assessment of validation rates became available, showing that >10× NGS coverage of a variant provides high confidence of its existence, and the several variants in Table 2 were not Sanger sequenced, as indicated. However, segregation studies in family members were always performed whenever samples were available, which showed 100% concordance with expected results, as shown (Table 2).

Five samples had mutations in *RHO*, making it the most commonly identified gene associated with pericentral RP, to date, across studies (see Table 3 and Discussion). No mutations were found in *TOPORS* or *ABCA4*, two genes identified in other studies (see Discussion.)

**Figure 2:**
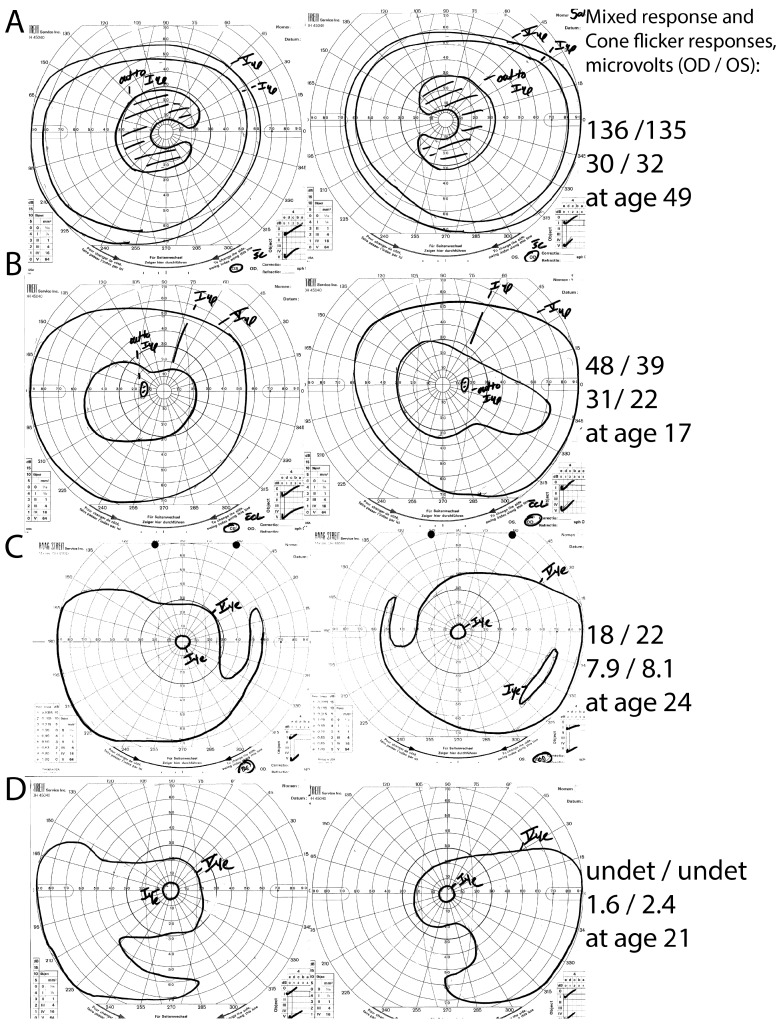
Intra-familial phenotypic variation in family #18. The proband (**A**) had pericentral RP, with near-peripheral C-shaped scotomas with preserved peripheral I4e responses, and relatively preserved full-field ERG responses. His children, in contrast, had either atypically mild RP without pericentral features (**B**) or typical RP (**C**,**D**). Note the constricted peripheral I4e stimulus responses and lower ERG response amplitudes in the children (**B**–**D**) in comparison to the proband. undet = undetectable.

Family #18 was the largest family available in this study, with a proband, his three affected children, and his unaffected wife. A *PRPF31* 3′ splice mutation (c.421-1G>A) was detected in all affected family members and was absent in the unaffected wife. Interestingly, while the proband had pericentral RP, his children with the same mutation had typical RP (*n* = 2) or mild RP without pericentral features (*n* = 1). See Figure 2.

Nine samples unsolved by panel-based NGS were analyzing using Omni2.5 chips (Illumina). No pathogenic CNVs were identified.

### 3.3. Whole Exome Sequencing Results

Previous studies used a candidate gene approach or panel-based sequencing to identify genetic causes of pericentral RP. To broaden this search for novel gene that cause pericentral RP, WES was performed on unsolved probands, and their family members, when available. From the probands not solved by panel-based NGS, 16 families were selected for WES. Three families had DNA available from multiple family members, while 13 were run as single samples.

A “burden test” was used to rank genes according to which genes had more damaging mutations in the pericentral set than in a control set (see Section 2.5 and Discussion.) Sixteen unsolved pericentral RP probands were compared to a set of 1724 exomes representing all other WES samples in our database. First, a dominant model was assumed in which only one damaging allele (i.e., a heterozygote mutation) was required to be counted. In each sample, there were a large number of genes contained “damaging” variants (as defined in Section 2.5); over 100 genes were implicated in every sample (range 117–233 not shown). This large number of hits makes it difficult to find the real solution, which is mostly likely a single gene in these Mendelian diseases/families. The top hits included a number of genes with large transcripts (e.g., *OBSCN* 24 kb mRNA, *HMCN1* 18 kb mRNA) with “damaging” alleles in >100 exomes in the control set, indicating that they are not likely to cause a rare disease. A more stringent test was performed which added a filter for ExAC allele frequency and omitted variants predicted as damaging by PolyPhen or SIFT. In this case, fewer positive hits were detected from each sample (range: 4–23 genes per sample, Supplemental Table S2), but still not few enough to identify meaningful solutions. Also, the list contains an overrepresentation of single base pair insertions (not shown), suggesting additional filtering strategies are required. Until these issues can be resolved, analyses of hits from the dominant inheritance model have been deferred.

Next, a recessive model was assumed which required two predicted-damaging variants (i.e., homozygous or compound heterozygous variants) in a sample for it to contribute to the gene count. Each sample contributed only 0–15 genes (average 6) under these conditions (Supplemental Table S3). The most enriched gene was *HGSNAT*, which was found in 3 of 16 unsolved pericentral RP samples (#34, #15, #13) and only 2 of 1726 other samples, *p* = 6 × 10^−6^ for enrichment, *p* = 6 × 10^−4^ with Bonferroni correction. One of the other samples was actually a pericentral sample provisionally considered solved (#24) by panel-based sequencing, making the enrichment even stronger. We categorized two of these samples as solved by *HGSNAT* mutations (#34 and #15), and two samples (#24, #13) with VUS in *HGSNAT* (Table 2). The latter two samples have homozygous mutations in a known, relatively common hypomorphic allele A615T, which has been reported to be pathogenic when combined with other alleles (see Discussion). Sample #15 contained a + 1 5’ splice variant that has been reported as pathogenic [43], in combination with the common A615T variant discussed below. Family members are not available for segregation testing. Sample #34 has a novel homozygous S318N mutation predicted to be damaging.

**Figure 3:**
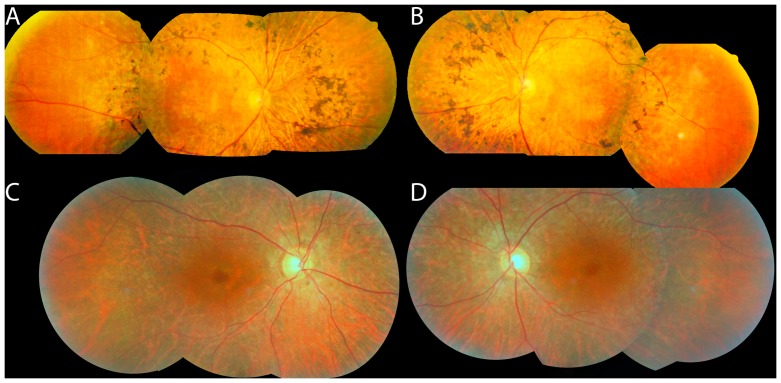
Fundus photos of patients with *HGSNAT* mutations: #34 (**A**,**B**) and #15 (**C**,**D**). Note that the bone spicules (**A**,**B**) or RPE depigmentation (**C**,**D**) are more posteriorly located than in typical RP, and that the fundus begins to show a more normal color peripherally (especially temporally in these photos).

*HGSNAT* mutations most commonly cause Mucopolysaccharidosis type IIIC, but review of the literature reveals nonsyndromic RP cases with what is essentially a pericentral phenotype (see Discussion). Fundus photographs of the two patients considered solved by *HGSNAT* mutations in this study are shown in Figure 3. Available records did not make any note of nonocular manifestations of mild HGSNAT deficiency, such as “coarse facial features, hypertrichosis, contractures, organomegaly, hearing impairment, behavioral and sleeping problems, recurrent infections, diarrhea, epilepsy or late onset of mental deterioration.” [44], p. 3745.

Additional enriched genes under the recessive model were not significant after multiple test correction.

In essence, burden testing analyses on the WES data successfully identified *HGSNAT* as a cause of pericentral RP, in at least two patients.

## 4. Discussion

### 4.1. What Genetics Reveals about the Causes of the Pericentral RP Phenotype

There is no specific genetic cause of pericentral RP. This study more than doubles the number of pericentral RP patients successfully genotyped. Yet there is still no single pericentral RP gene that explains most of the probands with this clinical phenotype. However, closer analysis of the genetic causes does give some flavor of what the genetic source of the phenotype is, and which other phenotypes are nearby to this phenotype in the sense that the causal genes overlap. Namely, the genetic causes of pericentral RP are similar to those that have been reported to cause other forms of mild RP. Grouping the three pericentral cohorts summarized in Table 3, *RHO* is the most commonly identified causative gene. *RHO* typically produces milder dominant disease [24,45,46,47] or sometimes minimally progressive sector RP [5,48]. We also identified one case as the X-linked RP/*RP2* carrier state [49], which also tends to produce a relatively mild phenotype compared to typical RP. About half of the gene variants described in this study have been previously reported in patients with typical RP. While it is possible that, on close inspection of the phenotype, all of these variants will be mild/hypomorphic mutations at a molecular level, it seems much more likely that there is broad overlap with the phenotypes of typical RP, atypically mild RP, and pericentral RP, even within a particular genotype. This overlap is demonstrated perfectly by family #18 in Figure 2 above where the same genotype causes typical RP, atypically mild RP, and pericentral RP. Cis-acting variants or modifier genes may explain some of this intra-familial diversity (e.g., [50]) and identifying additional modifier genes is an area of active research.

There are admittedly limitations to this interpretation of pericentral RP being caused by genes that typically produce mild disease; there are multiple recessive RP genes in Table 3, and some such as *USH2A* typically cause a large amount of field loss [47]. Furthermore, while pericentral RP is mild (by definition) in that there is a large amount of remaining visual field function as reflected by total visual field area and ERG responses, the better peripheral retinal function is accompanied by worse central/pericentral field and sometimes worse central acuity [19,22].

The genotypes identified in this study were most similar to those reported by Grondahl et al. [23] and Selmer et al. [25], where *RHO* was the most commonly identified gene. In contrast, as displayed in Table 3, Matsui et al. [19] found *ABCA4* as the most commonly identified gene in their “pericentral retinal degeneration” cohort. Matsui et al. carefully detail how the phenotypes of their five *ABCA4* cases are different from typical Stargardt disease with foveal sparing (e.g., no flecks, larger remaining central island of vision); nonetheless, subtle differences in inclusion criteria of mild, macula-predominant cases whose phenotype falls in between that of typical Stargardt disease and typical RP probably explain the differences observed genotypes.

### 4.2. Whole Exome Sequencing-Based Gene Discovery and *HGSNAT*

WES analysis strategies for this cohort are not trivial. Any WES dataset contains a large number of nonpathogenic variants. The inclusion of a control group for comparison helped to address this problem. Furthermore, some notable successes of WES-based gene discovery analyzed diseases where the genetic cause is limited to one or two genes (e.g., Kabuki syndrome [51]), and where the phenotype is very distinctive. For any cohort of RP patients, these advantages are not expected due to the large number of genes that can cause RP, ~50% diagnostic rates, and imperfections in clinically-defined phenotypic boundaries. Therefore, we assumed there would be heterogeneity in the results, decreasing the power to detect new genes with confidence. Given those challenges of WES-based cohort analyses, it is notable that WES did provide additional information in this cohort—the identification of *HGSNAT* as a recurring cause of pericentral RP.

Mutations in *HGSNAT* usually cause Sanfilippo Syndrome/Mucopolysaccharidosis Type IIIC—a severe multisystemic lysosomal storage disease that presents in infancy or childhood and leads to mental retardation, early death and, among other problems, RP [52]. More recently, *HGSNAT* mutations have been identified as a cause of nonsyndromic RP [44]. Haer-Wigman et al. [44] describe multiple patients with ring scotomas and one with a pericentral field defect. The images published in Figure 3G,H of that study show autofluorescence defects restricted to the near periphery [44]. Similarly, Van Cauwenbergh et al. [53] report on a patient with compound heterozygote mutations in *HGSNAT* (A615T/deletion of exons 7–8). While not explicitly described as a pericentral phenotype, Figure 2A of that study shows pigment changes, atrophy, and autofluorescence changes in the near periphery [53]. Fundus photographs of the two *HGSNAT* probands identified in the present study are shown in Figure 3, and they have a similar pericentral phenotype. (Unfortunately, those patients are no longer available for a more detailed phenotypic analysis such as enzyme levels.) Most recently, one additional case of *HGSNAT*-associated RP, with compound heterozygous A615T/P283L mutations, has been reported but without detailed phenotype information [54]. In summary, while there is mention of *HGSNAT*-associated nonsyndromic RP as having a perimacular or pericentral phenotype [44], this study serves to merge those observations with the larger body of literature about pericentral RP per se, and also suggests that *HGSNAT* is one of the more common genes to cause the pericentral RP phenotype. Practical implications of this finding are that *HGSNAT* should be included in panel-based testing of nonsyndromic RP, and evaluation for the reported [53] deletion of exons 7–8 should be considered in pericentral cases as well.

The pathogenicity status of the *HGSNAT* A615T allele appears to be complicated. This is particularly relevant to probands #13 and #24 in this study with homozygous A615T variants, but without another allele or detectable deletions. Our best interpretation of the existing data is that A615T is a weak mutation in that may cause no disease or mild disease in the homozygous state, but can cause mucopolysaccharidosis and/or RP when paired with a stronger allele [44,55,56,57]. The evidence supporting this interpretation is complex and not definitive. The A615T variant was originally identified as a cause of Mucopolysaccharidosis Type IIIC (described in that study as A643T) [55]. Feldhammer el al. [56] identify A615T as having slightly reduced activity, which was interpreted as wild type level in their original paper, but has been reinterpreted as a hypomorphic allele by Haer-Wigman et al. [44]. Haer-Wigman et al. [44] report an RP pedigree with heterozygous G133A mutations in combination with homozygous A615T variants—that is, G133A in *cis* with A615T on one allele, and A615T alone on the other allele. This suggests A615T can cause RP when paired with a stronger allele. Biochemically, a similar pattern has been reported. Fedele et al. [57] show that a combination of W403C and A615T in cis additively leads to lower activity, but that the A615T variant alone has a negligible decrease in activity; they state that A615T homozygotes probably would not be affected. Frequency data supports non-pathogenicity of homozygous A615T as well; the allele frequency for A615T appears particularly high in Ashkenazi Jews (1.5% with 2/4993 homozygotes reported [58]), such that this mutation is likely too common to be a rare cause of RP. Additional data such as evidence of partial penetrance or of high actual *HGSNAT*-associated disease incidence in Ashkenazi Jews could lead to reevaluation of this conclusion, however. Conversely, it is also not certain that every instance of compound heterozygote with A615T is disease-causing. For now, Table 2 lists homozygous A615T variants as a VUS, while compound heterozygotes with another pathogenic allele are listed as provisionally disease-causing.

In conclusion, these findings confirm and extend the observation that pericentral RP can be caused by many of the same mutations that cause typical RP, in a broad spectrum of genes that are known to cause typical RP. Additionally, the genotypes in the present cohort (e.g., *RHO*, *RP2* carrier state) suggest that pericentral RP shares genetic causes with other forms of mild RP. WES-based gene discovery analysis also allowed us to identify *HGSNAT* mutations as a recurring cause of pericentral RP. Out of the small number of reported cases of *HGSNAT*-associated RP, many appear to have pericentral features.

## Figures and Tables

**Table 1:** Baseline clinical characteristics of cohort probands. Further details regarding genes (last column) are provided in Table 2.

Family#	ID#	Sex	Age at First Visit (Years)	VA Snellen Equiv.	VA Decimal	ERG Combined Response Amplitude (µV)	ERG Cone Flicker Amplitude (µV)	V4e Total Field Area (deg²)	V4e Field Equivalent Diameter (deg)	V4e Field Description	Gene
1	003-292	F	42	20/20	1	158	38	12,793	128	ring scotoma to I4e OU	*PDE6B*
2	043-045	F	63	20/31	0.65	50	2	12,447 *	126 *	pericentral scotoma	
3	043-009	M	48	20/30	0.67	291	26	NA	NA	pericentral scotoma to V4e	
4	043-010	F	50	20/20	1	271	43	12,119	124	pericentral scotoma	*RHO*
5	043-011	M	29	20/27	0.74	114	44	NA	NA	constricted w mid-peripheral scotoma	*PDE6B*
6	043-012	F	67	20/20	1	32	6	4082	72	constricted with temporal crescents	*CNGA1*
7	043-013	F	58	20/25	0.8	341	53	NA	NA	full V4e OU; mid-peripheral scotoma I4e OU	*RHO*
8	043-014	M	41 *	20/31	0.65	136	34	9136	108	mid-peripheral scotoma	*RHO*
9	043-015	M	67	20/27	0.75	144	18	NA	NA	constricted OD; NA OS	
10	043-053	M	51	20/20	1	153	23	NA	NA	pericentral scotoma	
11	043-054	M	58	20/20	1	117	47	14,430	136	full V4e, pericentral scotoma I4e	
12	043-019	F	57	20/37	0.54	139	13	8368	103	pericentral scotoma	*USH2A*
13	231-023	M	35	20/20	1	180	40	13,083	129	full to V4e, pericentral scotoma to I4e	*HGSNAT?*
14	038-159	F	50 *	20/20	1	249	56	12,321	125	mid-peripheral scotoma	*RHO*
15	043-034	F	55	20/33	0.6	251	33	15,041	138	full to V4e, pericentral scotoma to I4e	*HGSNAT*
16	043-027	M	47	20/20	1	71	18	10,563	116	full v4e, constricted w pericentral scotoma I4e	
17	043-032	F	53	20/24	0.84	298	58	14,375	135	pericentral scotoma	*NR2E3*
18	043-043	M	46	20/20	1	182	27	14,867	138	full V4e, pericentral scotoma I4e	*PRPF31*
19	038-134	M	30	20/25	0.8	159	68	12,126	124	pericentral & mid-peripheral scotomas	
20	043-002	M	26	20/133	0.15	88	9	8064	101	constricted w peripheral islands	
21	043-003	F	78	20/54	0.37	229	26	108	12	constricted OU	
22	043-005	M	82	20/59	0.34	213	13	4868 *	79 *	pericentral scotoma	
23	043-006	M	50	20/22	0.9	106	33	NA	NA	constricted w peripheral islands	*RHO*
24	043-007	M	55	20/22	0.9	140	28	4700 *	77 *	constricted w scotoma OD; ring scotoma OS	*HGSNAT?*
25	043-008	F	83	20/71	0.28	59	2	NA	NA	constricted	
26	043-016	F	57 *	20/25	0.8	201	32	13,612	132	ring scotoma	*USH2A*
27	043-017	F	61	20/118	0.17	155	26	9673 *	111 *	constricted with ring scotoma V4e OU	
28	043-018	F	42	20/27	0.74	249	40	11,187	119	ring scotoma	
29	043-048	F	62	20/22	0.9	215	51	9392	109	mid-peripheral scotoma V4e, ring scotoma I4e	
30	043-049	F	46	20/20	1	125	46	12,594 *	127 *	full V4e, pericentral scotoma I4e	*USH2A*
31	043-056	F	49	20/20	1	172	19	13,369	130	paracentral nasal field loss	*RP2* carrier
32	043-057	F	45	20/31	0.65	167	26	13,296	130	pericentral scotoma	
33	043-058	F	62	20/34	0.59	155	47	14,262	135	constricted with ring scotoma OS, islands OD I4e	*TULP1*
34	038-162	M	63	20/333	0.06	134	14	9015	107	central and pericentral scotomas	*HGSNAT*
35	043-059	F	40	20/50	0.4	222	37	13,678	132	fairly full V4e; pericentral scotomas I4e OU	
36	043-060	M	51	20/20	1	192	35	10,008	113	ring scotoma to V4e OU	
37	043-061	M	49	20/20	1	186	45	12,850	128	pericentral scotoma	*USH2A*
38	043-062	M	44	20/20	1	138	34	13,327	130	pericentral scotoma	
39	043-063	F	55	20/25	0.8	236	43	13,546	131	pericentral scotoma	
40	043-067	M	26	20/20	1	86	34	8902	106	pericentral loss OU	*CNGB1*
41	043-068	M	71	20/20	1	116	10	13,466	121	bitemporal near mid-peripheral loss OU	
42	043-069	F	70	20/30	0.67	106	27	8551	104	pericentral field loss OU	
43	043-055	M	49	20/25	0.8	99	23	12,462	126	pericentral field loss OU	

M = male, F = female, VA = visual acuity, ERG = electroretinogram, µV = microvolts, NA = not available, deg = degrees, OU = both eyes, OD = right eye, OS = left eye. * Data not available at baseline; taken from subsequent visit.

**Table 2:** Genetic causes of disease in the pericentral RP cohort, including probands whose cause of disease was solved by panel-based sequencing (top) or whole exome sequencing (WES) (middle). Unsolved probands with VUS are shown below. Also, see Supplemental Table S1 for a more fully annotated version including genomic coordinates, pathogenicity predictions, and references.

Solved by Panel-Based Sequencing					
**Family#**	**Gene**	**Protein Variant**	**DNA Variant Description**	**Type**	**Sanger/Correct Segregation**
1	*PDE6B*	p.(Cys458Tyr)	NM_000283.3:c.1373G>A	HET	proband
1	*PDE6B*	p.(Lys518Ile)	NM_000283.3:c.1553A>T	HET	proband
4	*RHO*	p.(Gly101Val)	NM_000539.3:c.302G>T	HET	
5	*PDE6B*	p.(Gln298*)	NM_000283.3:c.892C>T	HET	proband
5	*PDE6B*	p.(Arg100His)	NM_000283.3:c.299G>A	HET	proband
6	*CNGA1*	p.(Thr586Serfs*17)	NM_000087.3:c.1755 _1758delAACT	HET	proband
6	*CNGA1*	p.(Ser320Phe)	NM_000087.3:c.959C>T	HET	proband
7	*RHO*	p.(Gly18Asp)	NM_000539.3:c.53G>A	HET	proband
8	*RHO*	p.(Thr58Arg)	NM_000539.3:c.173C>G	HET	proband
12	*USH2A*	p.(Glu3448Lys)	NM_206933.2:c.10342G>A	HOM	proband, het in unaffected son
14	*RHO*	p.(Gly106Arg)	NM_000539.3:c.316G>A	HET	proband
17	*NR2E3*	p.(Val118Met)	NM_014249.3:c.352G>A	HET	proband, affected sister
18	*PRPF31*	p.?	NM_015629.3:c.421-1G>A	HET	proband, 3 affected children
23	*RHO*	p.(Gly51Arg)	NM_000539.3:c.151G>C	HET	proband
26	*USH2A*	p.(Cys870*)	NM_206933.2:c.2610C>A	HET	proband
26	*USH2A*	p.(Asn42Lys)	NM_206933.2:c.126C>G	HET	proband
26	*USH2A*	p.(Gly2313Cys)	NM_206933.2:c.6937G>T	HET	proband
30	*USH2A*	p.?	NM_206933.2:c.9571-2A>G	HET	proband
30	*USH2A*	p.?	NM_206933.2:c.7595-3C>G	HET	proband
31	*RP2* carrier	p.(Gly163Glu)	NM_006915.2:c.488G>A	HET	proband
33	*TULP1*	p.?	NM_003322.3:c.1496-6C>A	HET	proband
33	*TULP1*	p.(Gln163*)	NM_003322.3:c.487C>T	HET	proband
37	*USH2A*	p.?	NM_206933.2:c.12067-2A>G	HET	proband
37	*USH2A*	p.(Cys3306Trp)	NM_206933.2:c.9918T>G	HET	proband
37	*USH2A*	p.(Ala1953Gly)	NM_206933.2:c.5858C>G	HET	proband
40	*CNGB1*	p.(Arg396Gln)	NM_001297.4:c.1187G>A	HET	
40	*CNGB1*	p.?	NM_001297.4:c.1801+5G>A	HET	
**Solved by Exome Sequencing**					
15	*HGSNAT*	p.(Ala615Thr)	NM_152419.2:c.1843G>A	HET	
15	*HGSNAT*	p.?	NG_009552.1(NM_152419.2):c.1464 + 1G>A	HET	
34	*HGSNAT*	p.(Ser318Asn)	NM_152419.2:c.953G>A	HOM	
**Not Solved or Partially Solved Due to Variants of Uncertain Significance (VUS)**					
3	*ROM1*	p.(Leu238Cysfs*78)	NM_000327.3:c.708delC	HET	
3	*COL11A1*	p.(Arg762Gln)	NM_001854.3:c.2285G>A	HET	
13	*HGSNAT*	p.(Ala615Thr)	NM_152419.2:c.1843G>A	HOM	
16	*USH2A*	p.(Leu1378Pro)	NM_206933.2:c.4133T>C	HET	proband, affected brother
16	*USH2A*	p.(Ser1369Leu)	NM_206933.2:c.4106C>T	HET	proband, affected brother
20	*TRPM1*	p.(Gln1161His)	NM_002420.5:c.3483G>C	HET	
20	*TRPM1*	p.(Ser157Phe)	NM_002420.5:c.470C>T	HET	
24	*HGSNAT*	p.(Ala615Thr)	NM_152419.2:c.1843G>A	HOM	
25	*MKS1*	p.(Thr423Ile)	NM_017777.3:c.1268C>T	HOM	
29	*BBS9*	p.(Pro419Ala)	NM_198428.2:c.1255C>G	HET	proband
29	*BBS9*	p.(Glu753Val)	NM_198428.2:c.2258A>T	HET	proband
42	*OPA1*	p.(Ala115Val)	NM_015560.2: c.344C>T	HET	

ExAC AF = allele frequency [38], HET = heterozygous, HOM = homozygous.

**Table 3:** Genes identified in patients with pericentral RP (this study), pericentral retinal degeneration [19], or pericentral retinal dystrophy [23,25], with numbers of probands solved by each gene.

Gene	This Study	Matsui et al.	Grondahl et al. and Selmer et al.	Total
*RHO*	5	1	3	9
*USH2A*	4			4
*HGSNAT*	2+			2
*PDE6B*	2			2
*CNGA1*	1			1
*CNGB1*	1			1
*NR2E3*	1	1		2
*PRPF31*	1			1
*RP2* carrier	1			1
*TULP1*	1			1
*ABCA4*		5		5
*CERKL*		3		3
*CRX*		1		1
*DHDDS*		1		1
*PROM1*		1		1
*PRPH2*		1		1
*TOPORS*			1	1
Total	22	14	4	37

## Supplementary Materials

The following are available online at www.mdpi.com/2073-4425/8/10/256/s1. Table S1: Genetic causes of disease in the pericentral RP cohort- fully annotated version. Table S2: Burden test results for dominant gene model. Table S3: Burden test results for recessive gene model.
